# Temporal dynamics of volatile fatty acids profile, methane production, and prokaryotic community in an *in vitro* rumen fermentation system fed with maize silage

**DOI:** 10.3389/fmicb.2024.1271599

**Published:** 2024-02-20

**Authors:** Rajan Dhakal, André Luis Alves Neves, Rumakanta Sapkota, Prabhat Khanal, Lea Ellegaard-Jensen, Anne Winding, Hanne Helene Hansen

**Affiliations:** ^1^Department of Veterinary and Animal Sciences, Production, Nutrition and Health, University of Copenhagen, Frederiksberg, Denmark; ^2^Department of Environmental Science, Aarhus University, Roskilde, Denmark; ^3^Faculty of Biosciences and Aquaculture, Nord University, Bodø, Norway

**Keywords:** fermentation kinetics, amplicon sequencing, Euryarchaeota, Firmicutes, linear relationship

## Abstract

Anaerobic *in vitro* fermentation is widely used to simulate rumen kinetics and study the microbiome and metabolite profiling in a controlled lab environment. However, a better understanding of the interplay between the temporal dynamics of fermentation kinetics, metabolic profiles, and microbial composition in *in vitro* rumen fermentation batch systems is required. To fill that knowledge gap, we conducted three *in vitro* rumen fermentations with maize silage as the substrate, monitoring total gas production (TGP), dry matter degradability (dDM), and methane (CH_4_) concentration at 6, 12, 24, 36, and 48 h in each fermentation. At each time point, we collected rumen fluid samples for microbiome analysis and volatile fatty acid (VFA) analysis. Amplicon sequencing of 16S rRNA genes (V4 region) was used to profile the prokaryotic community structure in the rumen during the fermentation process. As the fermentation time increased, dDM, TGP, VFA concentrations, CH_4_ concentration, and yield (mL CH_4_ per g DM at standard temperature and pressure (STP)) significantly increased. For the dependent variables, CH_4_ concentration and yield, as well as the independent variables TGP and dDM, polynomial equations were fitted. These equations explained over 85% of the data variability (*R*^2^ > 0.85) and suggest that TGP and dDM can be used as predictors to estimate CH_4_ production in rumen fermentation systems. Microbiome analysis revealed a dominance of Bacteroidota, Cyanobacteria, Desulfobacterota, Euryarchaeota, Fibrobacterota, Firmicutes, Patescibacteria, Proteobacteria, Spirochaetota, and Verrucomicrobiota. Significant temporal variations in Bacteroidota, Campylobacterota, Firmicutes, Proteobacteria, and Spirochaetota were detected. Estimates of alpha diversity based on species richness and the Shannon index showed no variation between fermentation time points. This study demonstrated that the *in vitro* fermentation characteristics of a given feed type (e.g., maize silage) can be predicted from a few parameters (CH_4_ concentration and yield, tVFA, acetic acid, and propionic acid) without running the actual *in vitro* trial if the rumen fluid is collected from similar donor cows. Although the dynamics of the rumen prokaryotes changed remarkably over time and in accordance with the fermentation kinetics, more time points between 0 and 24 h are required to provide more details about the microbial temporal dynamics at the onset of the fermentation.

## 1 Introduction

The rumen ecosystem consists of a vast array of anaerobic microbes, such as bacteria, archaea, protozoa, and fungi, which coexist in a symbiotic relationship with the host (Gruninger et al., 2019). The host provides an anaerobic chamber with the proper temperature (38–42°C) and buffering conditions to maintain the rumen fluid at the ideal pH (6.0–7.0) for microbial growth (Russell, 2009). During ruminal fermentation, microbes break down feeds that are indigestible by the host and convert them into volatile fatty acids (VFAs), CO_2_, CH_4_, NH_3_, H_2_, and heat. Ruminants obtain more than 70% of their energy for maintenance and growth from VFA and 50–90% of the protein requirements are met by microbial protein synthesis during ruminal fermentation (Ibrahim and Ingalls, 1972). Amongst the by-products of rumen fermentation, enteric CH_4_ has recently gained the most attention in the scientific community because ~18% of global anthropogenic CH_4_ emissions are ruminant-related (Mizrahi et al., 2021). This statistic emphasizes the urgency of the problem and the need to develop and validate amelioratory approaches that can efficiently reduce enteric greenhouse gas (GHG) emissions from ruminants. Research progress on methane mitigation includes various strategies such as feed compositional changes (Haque, 2018), probiotics (Dhakal et al., 2023), seaweed (Pandey et al., 2022), fat supplementation, ionophores, nitrates, and 3-nitrooxypropanol (Ban et al., 2021). These strategies have been shown to reduce enteric CH_4_ emissions from ruminants, but there is a pressing need to develop and validate more strategies. *In vitro* rumen fermentations hold great significance as they provide a faster, cheaper, and less labor-intensive alternative to *in vivo* studies. This makes it easier to screen larger and more diverse samples for the extensive validation of anti-methanogenic feeds. Compared to *in vivo* studies, *in vitro* rumen fermentations have fewer ethical constraints regarding animal use and handling.

Rumen microbes can be categorized into i) liquid-associated populations, ii) solid-associated populations, iii) epithelium-associated populations, and iv) eukaryote-associated populations (Zhou et al., 2015). In batch (continuously fed or closed) *in vitro* rumen fermentations, microbes can be classified into the same first two categories listed above and a third group of microbes that utilize the by-products of fermentation. The liquid-associated population digests rapidly fermentable carbohydrates such as sugar and starch. The solid-associated populations are attached to the rumen fiber mat and digest fiber fractions such as cellulose and hemicellulose. The third type of microbes uses the by-products of fermentation: degrading lactate, producing propionate and oxidizing formate whilst using CO_2_ and H_2_ as energy sources, and producing methane. Rumen microbes can also be broadly classified based on functional activities, such as cellulolytic, amylolytic, proteolytic, lipolytic, and methanogenic (Hungate, 1966). Despite the wealth of knowledge available on rumen microbial classification, little temporal dynamics data of the microbiome and by-product generation in *in vitro* fermentation exist, thereby impeding a more realistic depiction of the *in vivo* model (Onime et al., 2013; Kang et al., 2017; Wei et al., 2022).

Since *in vitro* rumen fermentation is usually performed for 24–96 h (Bogat and Brune, 2003; Choi et al., 2021; Sucu et al., 2022; Kim et al., 2023; Li et al., 2023; Maduro Dias et al., 2023), comparisons of *in vitro* rumen fermentation features and microbiome composition at shorter time intervals have not been conducted in previous trials, limiting the identification of optimal time points for sample collection that could be useful to understand the fermentation process, microbial dynamics, VFA, and CH_4_ production. Contrary to the previous studies cited, the aim of this study was to investigate the temporal dynamics of rumen bacterial and archaeal communities combined with CH_4_ and VFA production at 6, 12, 24, 36, and 48 h of incubation and develop mathematical models to predict the outcomes of fermentations when certain chemical results, such as dDM, VFA, and CH_4_, are not available. The implementation of such models can serve as a reliable alternative to traditional analytical chemical methods such as gas chromatography (GC), thereby reducing the need for laborious and costly experimental procedures. Therefore, the objectives of this study were 1) to quantify *in vitro* rumen fermentation parameters, such as CH_4_ and VFA production and microbial composition, at specified times from 6 to 48 h of incubation and 2) to develop a model to predict CH_4_ emissions from VFA concentrations and dDM.

## 2 Materials and methods

### 2.1 Chemical composition analysis

The maize silage used in the fermentations was collected in 2017 and treated as described by Dhakal et al. (2023). In short, the material was freeze-dried, ground through a 2-mm sieve, and stored until use. The dry matter content of maize silage was determined by drying the samples at 105°C for 8 h. The ash content was determined by burning the samples overnight at 520°C in a muffle furnace. Fiber analyzes of unfermented samples were performed using an ANKOM200 fiber analyzer for NDF and ADF (Van Soest et al., 1991; ANKOM Technology, 2017). Heat-stable alpha-amylase and sodium sulphite were used according to the ANKOM protocol. Crude protein (CP) was determined by Kjeldahl nitrogen content using the VELP Kjeldahl system (VELP Scientifica, New York City, NY, United States). The fiber composition, crude protein, and ash contents of the fermentation of maize silage (MS) are listed in Table 1.

**Table 1:** Chemical composition of maize silage.

DM%	NDF%	ADF%	ADL%	Crude protein %	Ash %
93.05	44.81	24.86	1.86	8.3	4.3

DM, Dry matter; NDF, Neutral detergent fiber; ADF, Acid detergent fiber; ADL, Acid detergent lignin.

### 2.2 *In vitro* fermentation

A 500-mg sample + 10 mg of maize silage was weighed into four 100-ml Duran® bottles as described by Vargas-Bello-Pérez et al. (2022). Each bottle was attached to an automatic *in vitro* gas production system (ANKOM^RF^ Technology, Macedon, NY, United States). The module was programmed to release accumulated gas pressure (250 ms vent opening) when the pressure inside the units reached 0.75 PSI above the ambient pressure. The gas production pressure was measured every 10 min and converted to milliliter of gas produced per gram of incubated substrate. More details on this method can be found in Vargas-Bello-Pérez et al. (2022).

A buffer medium was prepared (Supplementary Table S3) for *in vitro* fermentation and flushed with CO_2_ as described by Menke et al. (1979) for 2 h to ensure anaerobic conditions. The temperature of the buffer was maintained at 39°C prior to the addition of the rumen fluid. Further anaerobic conditions were ensured by adding a reduction agent, sodium sulphide, and sodium hydroxide 10 min before the addition of rumen fluid. The rumen contents, including liquid and particulates, were collected from two fistulated Jersey heifers owned by the Large Animal Hospital at the University of Copenhagen and licensed according to Danish law (authorisation nr.2012-15-2934-00648) before morning feeding and transported to the laboratory in preheated thermos bottles. The fistulated heifers were fed a maintenance-level diet consisting of *ad libitum* haylage for more than 6 weeks before the experiment (85% DM; 7.5 MJ of net energy for 20 liters of milk /kg DM (analyzed by EUROFINS Lab); 11% CP) and fasted for 12 h before collecting rumen fluid. The collected rumen fluid was filtered and gently squeezed to collect microbes attached to the feed particles through two cheesecloth layers and added to the buffer in a 1:2 ratio. A total of 90 mL of this inoculum (buffer and rumen fluid) was added to each bottle, after which the headspace of each bottle was flushed with CO_2_, capped with the module head, and incubated in a thermoshaker (Gerhardt, Königswinter, Germany) at 39.5°C for 48 h.

Three fermentation experiments were undertaken, each for 48 h, with four bottles being extracted at 6, 12, 18, 24, 36, and 48 h. The extracted bottles were placed in an ice bath to stop fermentation, and pH was measured in each bottle. Filtration was performed to collect the unfermented residual in a weighed ANKOM F57 filter bag (Ankom Technology, Macedon, NY, United States). The filtrate was collected and stored in a falcon tube (Sarstedt) at −20°C for VFA and microbial composition analysis. The filter bag containing the undigested residue was dried overnight at room temperature and thereafter dried for 2 h at 105°C in a forced air oven, cooled to ambient temperature in a desiccator, and weighed to determine degraded dry matter.

### 2.3 Methane and VFA analysis

The gas released from the bottles was collected in gas-tight bags (SKC, Flex Foil PLUS) to measure methane concentration. The methane content in the gas-tight bags was measured directly after different incubation time points using a gas chromatograph (GC) (Agilent 7820A GC, Agilent Technologies, Santa Clara, CA, United States). The GC was equipped with an HPPLOT Q column (30 m × 0.53 mm × 40 μm), and H_2_ was used as the carrier gas. The column flow rate was 5 mL/min, and the TCD detector was set to 250°C with a reference and makeup flow of 10 mL/min. A 250-μL gas sample was taken from each gasbag after the contents were mixed and manually injected into the GC and replicated samples from each gasbag. The run time was 3 min at an isothermal oven temperature of 50°C. Calibration curves were calculated using standards containing 1, 2.5, 5, 10, 15, and 25% CH_4_ in nitrogen (Mikrolab A/S, Aarhus, Denmark). Subsequently, the total methane concentration (% of collected gas) was calculated.

The samples were defrosted at room temperature prior to VFA analysis, and the rumen fluid, metaphosphoric solution (5:1), and crotonic acid, used as an internal standard, were combined and incubated for 30 min before centrifugation at 14,000 rpm for 10 min. A syringe filter with a particle size of 0.2 μm was used to filter the supernatant (MiniSart Syringe Filter, Sartorius). A 1-ml sample from the filter was retained in 2-ml GC vials and subjected to further analysis. The VFA was determined by gas chromatography (Nexis GC-2030, Shimadzu Scientific Instruments Inc., Kyoto, Japan) equipped with a 30-m wall-coated open-tubular fused-silica capillary column (Stabil-wax-DA; 30 m × 0.32 mm i.d., 0.25 μm film thickness; Shimadzu, United States). The run time per sample was 8.71 min. The oven temperature was programmed at 145°C for 3 min and then increased from 145°C to 245°C at 16.6°C/min. The injector and the flame ionization detector were maintained at 250°C. The gas flow rates were 24, 32, and 200 mL/min for N_2_, H_2_, and air, respectively. Crotonic acid was used as the internal standard, and a volatile-free acid (Sigma-Aldrich, St. Louis, MO, United States) was used as the external standard. Fiber analysis was carried out in the ANKOM200 fiber analyzer for NDF and ADF (Van Soest et al., 1991; ANKOM Technology, 2017).

### 2.4 DNA extraction

After defrosting the frozen samples, 2 mL of the fluid was placed in a fresh, clean tube and centrifuged at 15,000 rpm for 10 min to obtain genomic-rich pellets for genomic DNA extraction. Following the manufacturer’s instructions, DNA was extracted from cell-rich pellets using Bead-Beat Micro Ax Gravity (A&A Biotechnology, Gdynia, Poland). The concentration and purity of the extracted DNA were measured using a NanoDrop Lite UV–Vis spectrophotometer (Thermo Fisher Scientific).

### 2.5 16S rRNA gene amplicon sequencing

The prokaryotic primers 515F (GTGCCAGCMGCCGCGGTAA) and 806R (GGACTACHVGGGTWTCTAAT), along with Illumina Nextera overhang adapters, were used to amplify the V4 region of the bacterial 16S rRNA region (Caporaso et al., 2011). For the first PCR run, thermocycler conditions were 95°C for 2 min, 33 cycles of 95°C for 15 s, 55°C for 15 s, 68°C for 40 s; and final elongation at 68°C for 4 min (SimpliAmp Thermal Cycler, Applied Biosystems, California, USA). Each PCR reaction of 25 μL consisted of 5xPCRBIO HiFi Buffer (5 μL) m (PCR Biosystems, United Kingdom), 2 ng of DNA template, 0.25 unit of PCRBIO HiFi Polymerase (PCR Biosystems, UK), 0.5 mM of forward and reverse primers, 0.5 μL of bovine serum, and 16.25 μL of H20. A second PCR (PCR2) run was performed to add unique index combinations (i7 and i5) and adaptors. For PCR2, thermocycler conditions were 98°C for 1 min, 13 cycles of 98°C for 10 s, 55°C for 20 s, 68°C for 40 s, and a final elongation at 68°C for 5 min. Subsequently, the amplicon product was cleaned using HighPrep™ magnetic beads (MagBio Genomics Inc. Gaithersburg, USA) according to the manufacturer’s instructions. Finally, amplicon libraries were pooled in equimolar concentrations and sequenced using the Illumina MiSeq platform at Aarhus University.

### 2.6 Statistical and bioinformatics analysis

Statistical analyzes were performed in R version 4.2.1 (R Core Team, 2022).^1^ For all response variables, the following model was used to compare the means between different time points:

Yijk=μ+Ti+Rj+Eijk

where *Yijk* is the observation of the *ith* time (*Ti*), *μ* is the general mean, and *Rj* is the random effect of fermentation (three incubations). A linear mixed model was used to analyze differences at specified time points using the R function *lme* (Pinheiro and Bates, 2023).

MATLAB (2022) was used to generate the predictive equations. Polynomial fits were investigated with the data, and the curve with the lowest root mean squared error (RMSE) for each response was used to select the best model for the fitted curve.

Using QIIME2 and the DADA2 plugin (Callahan et al., 2016), the DNA reads acquired from the Illumina MiSeq run were analyzed (Bolyen et al., 2019). The DADA2 plugin in QIIME2 was used to screen for chimeras after denoising, joining, dereplicating, trimming forward and reverse primers, and building an amplicon sequence variant (ASV) table. ASVs were then assigned a taxonomy using the SILVA 138 database and the ‘feature-classifier classify-sklearn’ method (Quast et al., 2013). The taxonomy files and ASV table were imported into R version 4.2.1 for data analysis and visualization (R Core Team, 2022). The *vegan* package version 2.6–4 and the *phyloseq* package version 1.40.0, by Oksanen et al. (2020) and McMurdie and Holmes (2013) were used for diversity-based analysis.

## 3 Results

### 3.1 Fermentation kinetics/rumen fermentation characteristics

As the fermentation time increased, degraded dry matter (dDM), total gas production (TGP), methane (CH_4_) concentration and yield, and total volatile fatty acid (tVFA) and volatile fatty acid (VFA) composition increased (*p* < 0.0001), as shown in Table 2. However, no significant differences (*p* > 0.05) were observed between 36 and 48 h in dDM, TGP, CH_4_ yield, and total VFA. Similarly, no differences (*p* > 0.05) in CH_4_, propionic acid, isobutyric acid, and butyric acid concentrations were observed between 24 h, 36 h, and 48 h. However, a difference (*p* < 0.05) was observed at 48 h of incubation for isovaleric acid and caproic acid.

**Table 2:** Effect of time on dDM, TGP, CH_4_ (concentration and yield), and VFA composition during 48 h of maize silage *in vitro* fermentation.

Items	6 h	12 h	24 h	36 h	48 h	SEM	*p*-value
dDM	8.27^d^	26.44^c^	56.03^b^	66.05^a^	70.5^a^	1.99	<0.0001
TGP/g DM	33.9 ^a^	95.8^c^	188.4^b^	213.7^a^	228.5^a^	9.69	<0.0001
CH_4_% of TGP	1.21 ^d^	4.72^c^	9.49 ^b^	10.16^b^	11.03^a^	0.917	<0.0001
CH_4_ ml /g DM	0.242^d^	5.366^c^	17.998^b^	21.844^a^	25.30^a^	2.06	<0.0001
Acetic acid (mMol/L)	14.9^e^	17.5^d^	22.8^c^	25.1^b^	26.80^a^	1.48	<0.0001
Propionic acid (mMol/L)	3.87^b^	4.53^b^	6.52^a^	6.63^a^	6.87^a^	0.429	<0.0001
Isobutyric acid (mMol/L)	0.216^c^	0.249^c^	0.328^b^	0.352^ab^	0.396^a^	0.0294	<0.0001
n-Butyric acid (mMol/L)	1.84^c^	2.76^b^	3.89^a^	3.99^a^	4.17^a^	0.349	<0.0001
Isovaleric acid (mMol/L)	0.277^c^	0.334^c^	0.453^b^	0.506^b^	0.594^a^	0.0666	<0.0001
Caproic acid (mMol/L)	0.105 ^b^	0.14^ab^	0.158^ab^	0.167^ab^	0.205^a^	0.0236	<0.0001
Total VFA (mMol/L)	21.5^d^	25.9^c^	34.8^b^	37.1^ab^	39.5^a^	1.46	<0.0001

a, b, c Values within a row are different if superscript differs (*p* < 0.05), dDM; degraded dry matter, TGP, total gas production at standard temperature and pressure; CH_4_%, methane concentration; CH_4_ ml, methane yield/g dry matter (DM).

The predictive equations for CH_4_ concentration (%) and yield, acetic acid, propionic acid, n-butyric acid, and t-VFA are shown in Table 3. Except for propionic acid (66.91%) and n-butyric acid (70.58%), other predictive equations explained more than 80% of the variation. The most precise model to predict methane yield had TGP (R^2^ = 0.9119), followed by dDM (*R*^2^ = 0.87) and time (h) (*R*^2^ = 0.85) as dependent variables.

**Table 3:** Predictive equation for CH_4_ concentration (%) and yield, acetic acid, propionic acid, n-butyric acid, and tVFA from parameters of hours, TGP, and dDM.

X	Y	Equation	R-squared	RMSE
Hours	CH_4_ ml	Y = − 0.0132*x^2 + 1.31*x − 7.412	0.8527	4.0615
Hours	CH_4_%	Y = 0.0002*x^3–0.0262*x^2 + 1.0583*x – 4.2813	0.8226	1.7642
TGP	CH_4_ ml	Y = − 0.0001*x^2 + 0.0673*x − 1.0749	0.9119	1.231
dDM	CH_4_ ml	Y = − 0.0028*x^2 + 0.0907*x – 0.0119	0.8737	0.0914
Hours	acetic acid	Y = − 0.0059*x^2 + 0.6*x + 11	0.8081	2.327
Hours	Propionic	Y = − 0.0027*x^2 + 0.2149*x + 2.59	0.6691	0.893
Hours	n-butyric	Y = 0.0001*x^3–0.0077*x^2 + 0.2916*x + 0.3249	0.7058	0.6196
Hours	tVFA	Y = −0.0110 *x^2 + 1.0151*x + 15.7164	0.8892	2.556

TGP, total gas production at standard temperature and pressure; VFA, volatile fatty acid, RMSE: root mean square error; dDM: degraded dry matter.

### 3.2 16S rRNA gene amplicon sequencing

Illumina MiSeq amplicon sequencing generated 723,348 reads, consisting of 6,915 amplicon sequence variants (ASVs). After filtration for quality control and removal of chloroplast and mitochondria reads, the total number of sequences was reduced to 723,304 with 3,694 ASVs. The average counts per sample assigned to ASV (post-filtering) were 24,112 ± 9,780.

### 3.3 Alpha diversity

**Figure 1:**
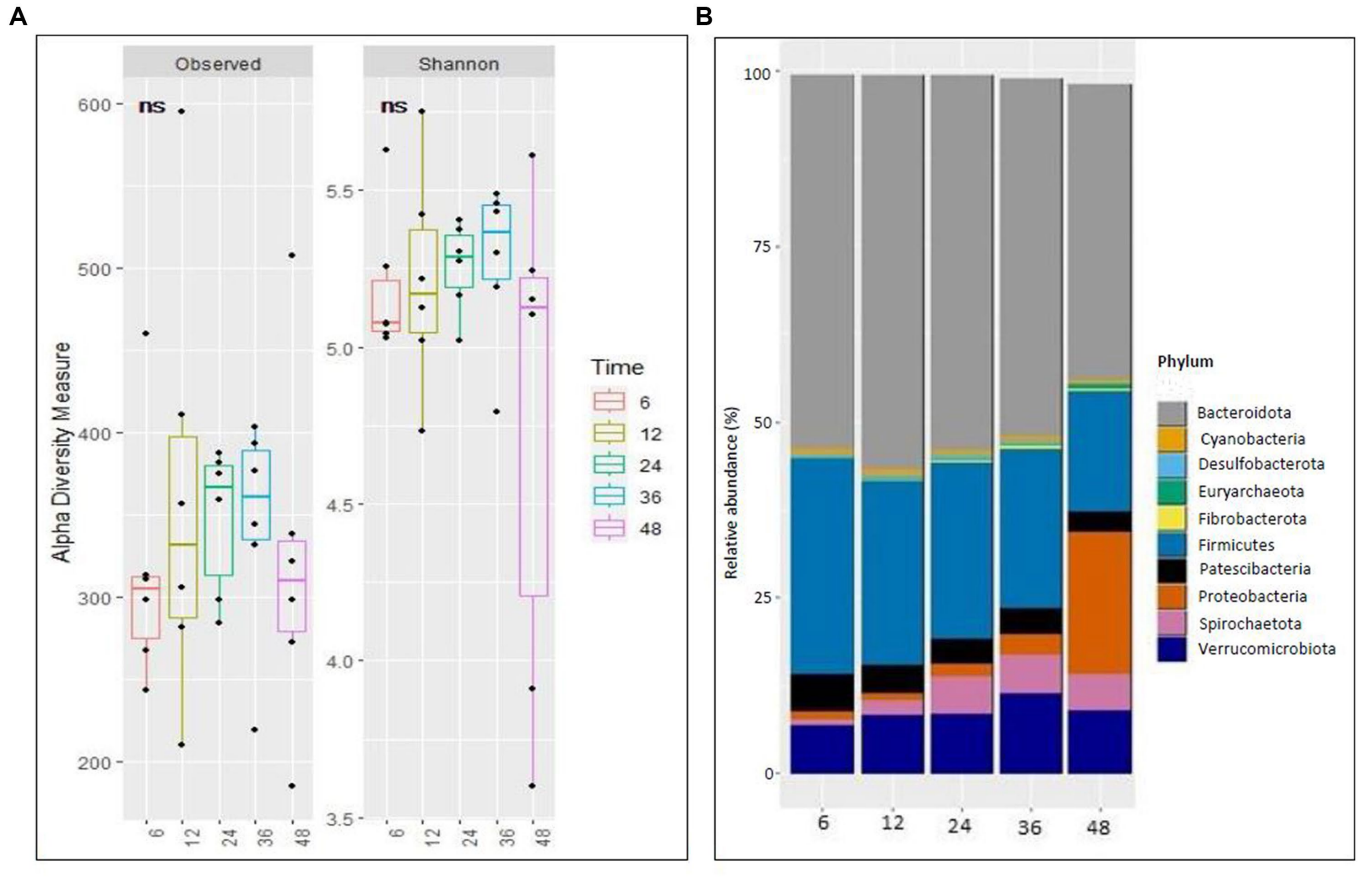
Richness and microbial composition across time points during fermentation. (A) Alpha (Observed richness and Shannon index) at ampliconsequence variants (ASVs) level and (B) Stacked bar plot illustrating the relative abundance of microbial phyla at different time points.

**Figure 2:**
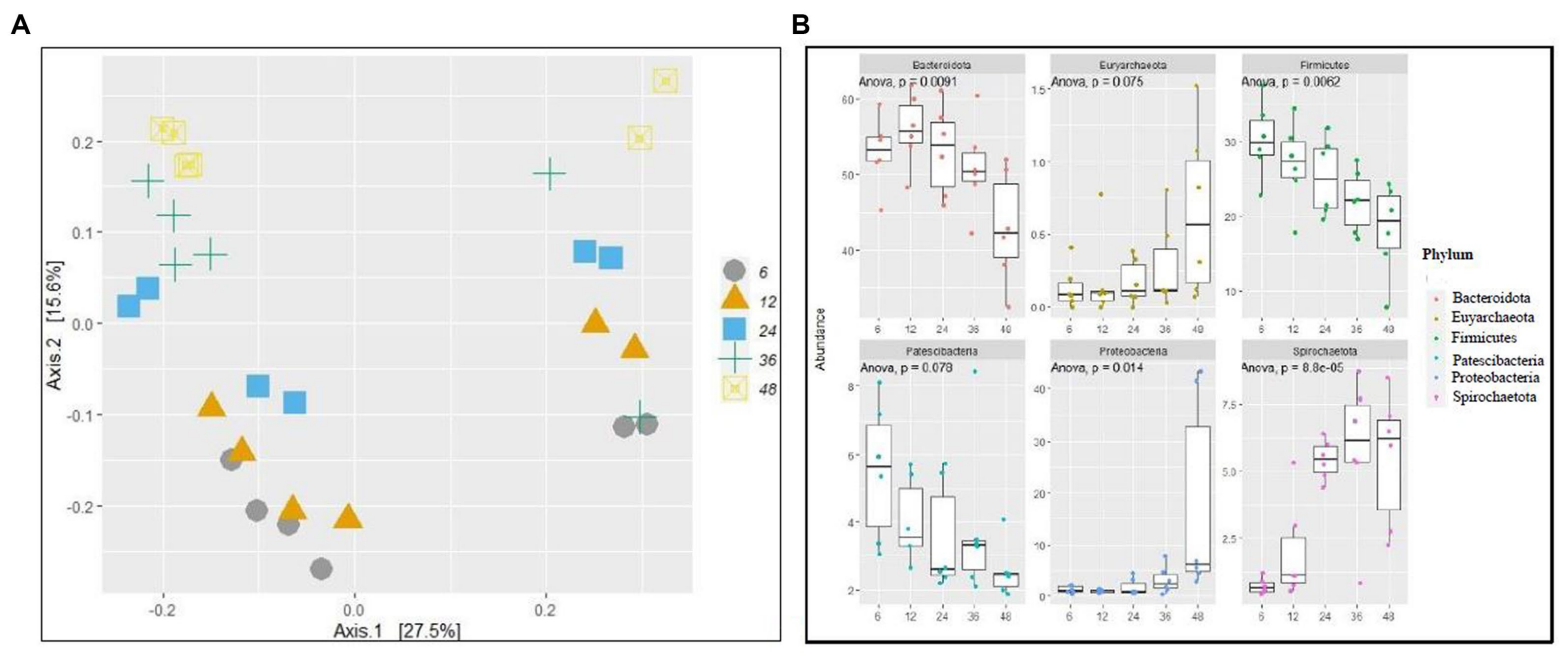
Fermentation time affects bacterial community structure. **(A)** principal coordinate analysis (PCOA) of prokaryotic beta-diversity based on Bray–Curtis dissimilarity distances and **(B)** relative abundance of a selected phylum of different time points of fermentations.

No difference (*p* > 0.05) was observed in alpha diversity using the Shannon index (6 h,5.18,12 h:5.21, 24 h:5.25, 36 h:5.26, 48 h:4.77) and species richness (observed) (6 h,316.5,12 h:359.39, 24 h:346.33, 36 h:342.33, 48 h:320.75) between the different fermentation time points (Figure 1A). Taxonomic analyzes at the phylum level of relative abundance are shown in Figure 1B and Supplementary Table S1. The phyla with an average > 1% across all samples were Bacteroidetes (51.2%), Firmicutes (24.58%), Verrucomicrobiota (8.71%), Proteobacteria (4.93), Patescibacteria (3.88), and Spirochaetota (3.88). The Bray-Curtis distance matrices were visualized using principal coordinate analysis revealing clustering based on fermentation time points (Figure 2A). The PERMANOVA test revealed a significant difference (*p* < 0.05) between the prokaryotic community structures at different time points. The abundances of Bacteroidetes, Firmicutes, Patescibacteria, Proteobacteria, and Spirochaetota at the phylum level were significantly different (*p* < 0.05) between different time points (Figure 2B).

### 3.4 Correlation

**Figure 3:**
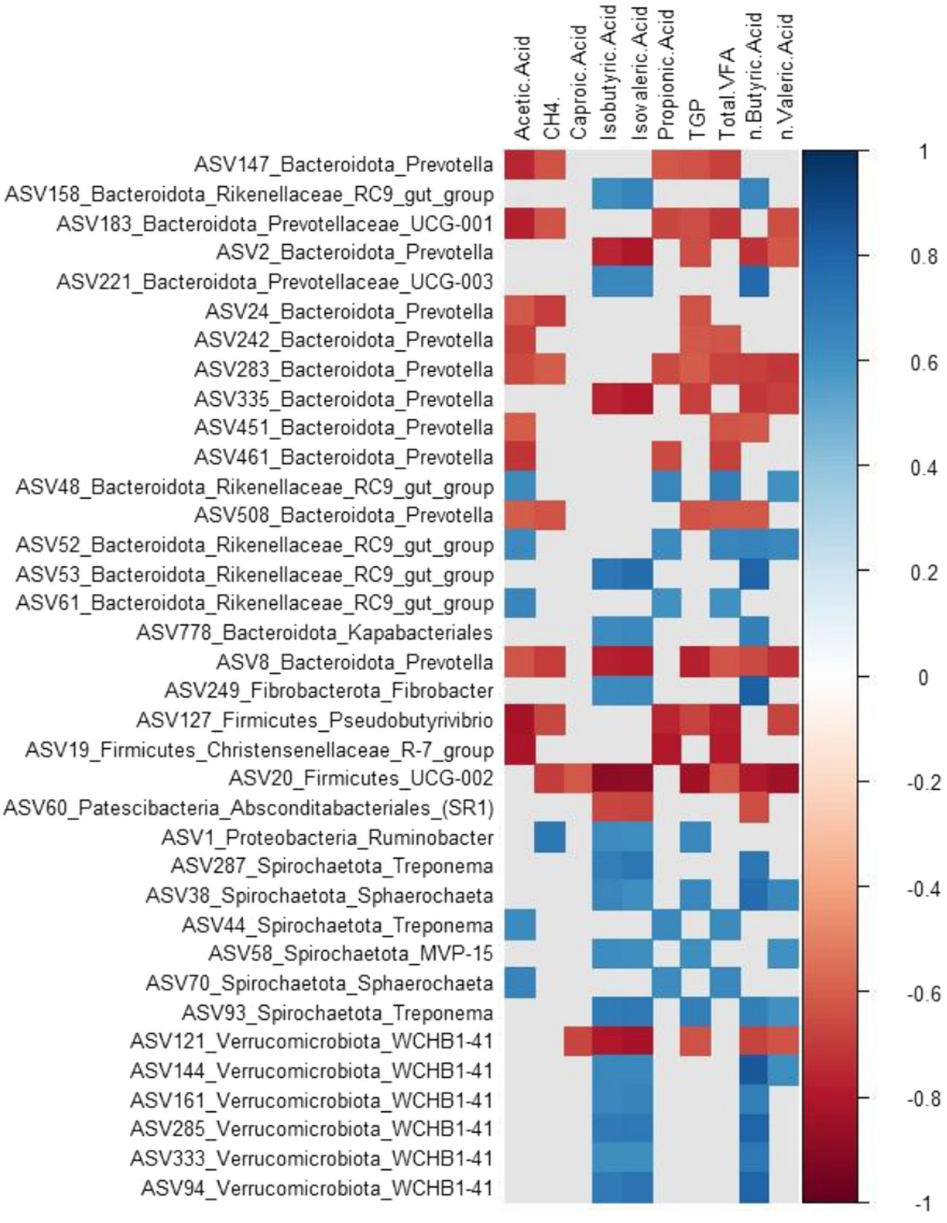
Microbial ASV-fermentation parameter correlation. Heat map showing spearman correlation among the microbial composition and fermentation parameters; CH4, methane concentration (%); TGP, total gas production; Total.VFA, total volatile fatty acid. Strong correlation (−0.6 > r > 0.6 and *p* < 0.05) were visualized.

Spearman’s correlation revealed both positive and negative interactions between the relative abundances of prokaryotic microbes and fermentation parameters (Figure 3; Supplementary Table S2). An association was considered strong when the correlation coefficient value was above 0.6 or weak when below 0.6, with the adjusted *p*-value being <0.05 to confirm the statistical significance of the correlation. The relative abundance of *Prevotella* (Bacteroidetes) was negatively correlated with acetic acid, CH_4_, isobutyric, isovaleric, propionic, TGP, tVFA, n-butyric, and n-valeric acid. Conversely, the relative abundance of *Rikenellaceae* (Bacteroidetes) was positively correlated with acetic acid, isobutyric, isovaleric, propionic, total VFA, n-butyric, and n-valeric acids. *Pseudobutyrivibrio* (Firmicutes) was negatively correlated with propionic acid, tVFA, n.valeric acid, TGP, and CH_4_%. In contrast, *Ruminobacter* (Proteobacteria) was positively correlated with CH_4_%, isobutyric, isovaleric, and TGP, and the relative abundance of *Treponema, Sphaerochaeta*, and *Spirochaetota_MVP-15* (Spirochaetota) was positively correlated with TGP and acetic, isobutyric, isovaleric, propionic, butyric, and valeric acids. Similarly, the relative abundance of *WCHB1-41* (Verrucomicrobiota) was positively correlated with isobutyric, isovaleric, and n-butyric acids. However, *ASV121_Verrucomicrobiota_WCHB1-41* correlated negatively with caproic, isobutyric, isovaleric, TGP, n-butyric, and valeric acids.

## 4 Discussion

The *in vitro* rumen fermentation approach precisely simulates *in vivo* rumen fermentation by assessing many samples in a batch at the same time, thereby decreasing the costs of animal care and related animal welfare concerns. This technique allows researchers to have better control over the research environment and eliminate host-related factors that might affect the fermentation process, such as the rumen passage rate and absorption. Different parameters, such as TGP, VFA, CH_4_ concentration and yield, dDM, and microbiome composition after fermentation, can be measured *in vitro* (Sato et al., 2020; Dhakal et al., 2022, 2023; Vargas-Bello-Pérez et al., 2022). The dynamics of dDM, TGP, CH_4_ concentration, and yield in this study were linear with time up to 36 h, and beyond that, no variation was observed, suggesting that a fermentation with a duration over 36 h is not required. As fermentation progresses, fiber breakdown increases, resulting in an increase in TGP, dDM, tVFA, and individual VFA (Eun et al., 2007; Wei et al., 2022). However, the kinetics of VFA were different: propionic and n-butyric acid increased by 24 h, whereas acetic acid and tVFA followed a linear pattern over time. Such an outcome can be attributed to the inherent nature of highly fermentable carbohydrates in maize silage, which tend to undergo rapid fermentation, resulting in a higher accumulation of propionic and butyric acids (Makkar, 2009). In this study, we observed rapid production of acetic acid and TGP between 12 and 36 h. We observed a higher yield of CH_4_ during this period, and this could be due to rapid acetic acid and CO_2_ production. In rumen fermentation, acetate production produces hydrogen, and propionic acid production consumes hydrogen (Ungerfeld, 2020). Therefore, the production of acetate favors the production of CH_4_, as archaea utilizes hydrogen and carbon dioxide to produce methane (Newbold and Ramos-Morales, 2020). This study provides evidence that in order to understand fermentation kinetics and its by-products in readily fermentable feedstuffs, such as maize silage, shorter time intervals at the beginning of the fermentation must be considered, with 12 h to 36 h being crucial time points.

In addition, we fitted polynomial equations that can be used to predict tVFA and acetic, propionic, n-butyric acids, dDM, and methane concentrations over time. Over the last 50 years, various mathematical models have been developed to predict CH_4_, VFA, and TGP from *in vitro* rumen fermentations (Moss et al., 2000; Getachew et al., 2002; Hegarty and Nolan, 2007; Alemu et al., 2011). The previous models predicting fermentation products often yield low *R^2^* values through regression, failing to capture the non-linearity of the data. However, we have improved upon these models by incorporating polynomial equations that can account for temporal changes and better fit the non-linearities of the fermentation data under consideration with a high *R^2^* (> 0.70). Our predictive polynomial equations have several potential applications, such as estimating methane generated from *in vitro* fermentations, offering a quick and easy way to evaluate feed and accelerate the development of anti-methanogenic compounds.

Prokaryotic alpha diversity explains species richness and evenness, which are measured using the observed and Shannon indices. Although alpha diversity did not vary over time in this study, the relative abundance of the core microbiome and prokaryotic beta diversity did. Bacteroidetes and Firmicutes were the dominant phyla at each time point, as supported by previous studies (Mizrahi et al., 2021; Zhao et al., 2023). In this study, Bacteroidetes, Firmicutes, Patescibacteria, Proteobacteria, and Spirochaetota exhibited dynamic changes over time. Bacteroidetes and Firmicutes are the two most dominant in the rumen, representing a major part of the core rumen microbiome (Jose et al., 2020; Mizrahi et al., 2021; Liang et al., 2022). The relative abundances of Firmicutes and Patescibacteria decreased over time, whereas Bacteroidetes increased until 12 h, Spirochaetota increased until 36 h, and Euryarchaeota and Proteobacteria increased over time. The substrate quantity used in our *in vitro* fermentation is limited to 0.5 g, meaning that this amount cannot be replenished because the system is closed. The substrate disappearance after a few hours of incubation decreases the population of microbes that depend on cellulose, hemicellulose, starch, and easily soluble carbohydrates, unlike the *in vivo* degradation of substrates, which is a continuous process. The decrease in the relative abundance of the core prokaryotic community over time using maize silage as substrate shows that i) the time points to be investigated are crucial to sample and interpret the data and ii) the results may lead to wrong conclusions if 48 h or longer fermentation time intervals are considered. If the overall dry matter digestibility of feedstuffs in the rumen is approximately 50% under optimum conditions, the best time to end *in vitro* fermentations will be after 18–24 h of incubation. The ecological niches of microbial fermentation change between time points depending on the available substrate, resulting in significant differences in the diversity and community structure of the microbiome. Pinnell et al. (2022) concluded that different ecological niches within each microenvironment resulted in significant differences in the diversity and community structure of microbial communities in rumen fluid without the influence of diet.

In this study, the association between the microbiome and fermentation parameters varied at the ASV level, regardless of phylum. Previous studies (Shen et al., 2017; Chang et al., 2021; Wei et al., 2022) have shown that Bacteroidetes, Firmicutes, Proteobacteria, Spirochaetota, and Verrucomicrobiota have a strong relationship with fermentation parameters. Our study also found similar results, although our treatments differed from those used in previous studies. We also observed changes in hydrogen-utilizing microbes such as “Euryarchaeota” depending on the fermentation by-products and time. It is challenging to discuss the role of Bacteroidetes, Proteobacteria, and Firmicutes in the production of VFA because multiple microbes with different pathways compete for the same substrates, which may lead to the competitive exclusion of other functional groups (Moraïs and Mizrahi, 2019). Although Bacteroidetes and Firmicutes are believed to be involved in propionate production (Henderson et al., 2015), our study found that these phyla showed both positive and negative correlations with propionate and other metabolites. This demonstrates that there is redundancy or overlapping distribution of physiological capabilities across multiple microbial taxa (Weimer, 2015).

## 5 Conclusion

Based on the results of tVFA and VFA composition, microbiome, CH_4_ yield, and concentration, this study provides evidence that sampling time points from 12 h to 36 h are crucial and must be considered to understand kinetics, by-product formation, and microbial dynamics in *in vitro* rumen fermentation batch systems fed highly digestible feedstuffs such as maize silage. We showed that microbiome changes and fermentation parameters can be predicted on a temporal basis using *in vitro* rumen fermentation characteristics and polynomial equations. The study was limited due to the choice of only one type of feed, which allowed a clear focus on the temporal changes of microbiome changes and fermentation parameters in a common dairy cattle feed. In order to obtain a more accurate and encompassing representation of the temporal dynamics of the rumen microbes, future research should include multiple feed and diet types, differing rumen fluid pH and donor age, as well as the stage of lactation.

## Data availability statement

The datasets presented in this study can be found in online repositories. The names of the repository/repositories and accession number(s) can be found at: https://www.ncbi.nlm.nih.gov/, PRJNA1000955.

## Author contributions

RD: Conceptualization, Data curation, Formal analysis, Funding acquisition, Investigation, Methodology, Project administration, Resources, Software, Visualization, Writing – original draft. AN: Formal analysis, Methodology, Software, Writing – review & editing. RS: Data curation, Formal analysis, Software, Validation, Writing – review & editing. PK: Writing – review & editing. LE-J: Methodology, Writing – review & editing. AW: Supervision, Writing – review & editing. HH: Conceptualization, Funding acquisition, Methodology, Project administration, Resources, Supervision, Writing – review & editing.

## Footnotes

^1^ https://www.R-project.org/
