# Supplementary material for: Temporal dynamics of volatile fatty acids profile, methane production, and prokaryotic community in an in vitro rumen fermentation system fed with maize silage
Source: Front Microbiol. 2024 Feb 20;15:1271599. doi: 10.3389/fmicb.2024.1271599 (PMC10912478; doi:10.3389/fmicb.2024.1271599)
Supplement: Supplementary file 1 [file Table_1.DOCX]

**Supplementary Table 1:** Relative abundance of phylum more than 0.5%

| **Abundance** | **6 h** | **12 h** | **24 h** | **36 h** | **48 h** | **Average** |
| --- | --- | --- | --- | --- | --- | --- |
| Bacteroidota | 53.02 | 55.94 | 53.26 | 50.97 | 42.97 | 51.23 |
| Firmicutes | 30.30 | 27.05 | 25.26 | 22.05 | 18.23 | 24.58 |
| Verrucomicrobiota | 6.80 | 7.57 | 8.31 | 11.17 | 9.74 | 8.718 |
| Patescibacteria | 5.50 | 4.03 | 3.49 | 3.84 | 2.55 | 3.883 |
| Proteobacteria | 1.29 | 0.99 | 1.76 | 3.179 | 17.45 | 4.934 |
| Cyanobacteria | 0.87 | 0.64 | 0.49 | 0.68 | 0.352 | 0.608 |
| Spirochaetota | 0.72 | 1.97 | 5.42 | 5.79 | 5.49 | 3.878 |
| Desulfobacterota | 0.49 | 0.52 | 0.66 | 0.371 | 0.210 | 0.450 |
| Euryarchaeota | 0.13 | 0.18 | 0.17 | 0.2771 | 0.654 | 0.283 |
| Campilobacterota | 0.001 | 0 | 0.009 | 0.237 | 1.03 | 0.255 |
| <0.5% | 0.06 | 0.079 | 0.083 | 0.103 | 0.094 | 0.084 |

**Supplementary Table 2**. Correlation between ASV and fermentation parameters.

|  | Acetic | CH_4_% | Caproic | Isobutyric | Isovaleric | Propionic | TGP | T-VFA | n.Butyric | n.Valeric |
| --- | --- | --- | --- | --- | --- | --- | --- | --- | --- | --- |
| ASV147_Bacteroidota_Prevotella | -0.76 | -0.63 | NA | NA | NA | -0.62 | -0.63 | -0.68 | NA | NA |
| ASV158_Bacteroidota_Rikenellaceae_RC9_gut_group | NA | NA | NA | 0.61 | 0.67 | NA | NA | NA | 0.65 | NA |
| ASV183_Bacteroidota_Prevotellaceae_UCG-001 | -0.77 | -0.62 | NA | NA | NA | -0.66 | -0.65 | -0.71 | NA | -0.64 |
| ASV2_Bacteroidota_Prevotella | NA | NA | NA | -0.76 | -0.81 | NA | -0.64 | NA | -0.72 | -0.62 |
| ASV221_Bacteroidota_Prevotellaceae_UCG-003 | NA | NA | NA | 0.64 | 0.65 | NA | NA | NA | 0.77 | NA |
| ASV24_Bacteroidota_Prevotella | -0.61 | -0.70 | NA | NA | NA | NA | -0.63 | NA | NA | NA |
| ASV242_Bacteroidota_Prevotella | -0.68 | NA | NA | NA | NA | NA | -0.62 | -0.63 | NA | NA |
| ASV283_Bacteroidota_Prevotella | -0.65 | -0.61 | NA | NA | NA | -0.65 | -0.61 | -0.67 | -0.69 | -0.70 |
| ASV335_Bacteroidota_Prevotella | NA | NA | NA | -0.77 | -0.79 | NA | -0.68 | NA | -0.70 | -0.68 |
| ASV451_Bacteroidota_Prevotella | -0.60 | NA | NA | NA | NA | NA | NA | -0.62 | -0.61 | NA |
| ASV461_Bacteroidota_Prevotella | -0.72 | NA | NA | NA | NA | -0.66 | NA | -0.68 | NA | NA |
| ASV48_Bacteroidota_Rikenellaceae_RC9_gut_group | 0.64 | NA | NA | NA | NA | 0.65 | NA | 0.68 | NA | 0.61 |
| ASV508_Bacteroidota_Prevotella | -0.60 | -0.63 | NA | NA | NA | NA | -0.62 | -0.61 | -0.61 | NA |
| ASV52_Bacteroidota_Rikenellaceae_RC9_gut_group | 0.63 | NA | NA | NA | NA | 0.63 | NA | 0.66 | 0.67 | 0.64 |
| ASV53_Bacteroidota_Rikenellaceae_RC9_gut_group | NA | NA | NA | 0.72 | 0.76 | NA | NA | NA | 0.81 | NA |
| ASV61_Bacteroidota_Rikenellaceae_RC9_gut_group | 0.65 | NA | NA | NA | NA | 0.60 | NA | 0.61 | NA | NA |
| ASV778_Bacteroidota_Kapabacteriales | NA | NA | NA | 0.62 | 0.64 | NA | NA | NA | 0.68 | NA |
| ASV8_Bacteroidota_Prevotella | -0.62 | -0.70 | NA | -0.77 | -0.78 | NA | -0.77 | -0.62 | -0.66 | -0.72 |
| ASV249_Fibrobacterota_Fibrobacter | NA | NA | NA | 0.63 | 0.64 | NA | NA | NA | 0.82 | NA |
| ASV127_Firmicutes_Pseudobutyrivibrio | -0.82 | -0.67 | NA | NA | NA | -0.76 | -0.68 | -0.78 | NA | -0.67 |
| ASV19_Firmicutes_Christensenellaceae_R-7_group | -0.82 | NA | NA | NA | NA | -0.80 | NA | -0.79 | NA | NA |
| ASV20_Firmicutes_UCG-002 | NA | -0.69 | -0.61 | -0.89 | -0.89 | NA | -0.83 | -0.61 | -0.79 | -0.85 |
| ASV60_Patescibacteria_Absconditabacteriales_(SR1) | NA | NA | NA | -0.67 | -0.67 | NA | NA | NA | -0.65 | NA |
| ASV1_Proteobacteria_Ruminobacter | NA | 0.72 | NA | 0.62 | 0.61 | NA | 0.65 | NA | NA | NA |
| ASV287_Spirochaetota_Treponema | NA | NA | NA | 0.69 | 0.73 | NA | NA | NA | 0.73 | NA |
| ASV38_Spirochaetota_Sphaerochaeta | NA | NA | NA | 0.66 | 0.62 | NA | 0.65 | NA | 0.77 | 0.65 |
| ASV44_Spirochaetota_Treponema | 0.64 | NA | NA | NA | NA | 0.64 | NA | 0.64 | NA | NA |
| ASV58_Spirochaetota_MVP-15 | NA | NA | NA | 0.62 | 0.62 | NA | 0.62 | NA | NA | 0.60 |
| ASV70_Spirochaetota_Sphaerochaeta | 0.67 | NA | NA | NA | NA | 0.62 | NA | 0.65 | NA | NA |
| ASV93_Spirochaetota_Treponema | NA | NA | NA | 0.71 | 0.72 | NA | 0.68 | NA | 0.69 | 0.60 |
| ASV121_Verrucomicrobiota_WCHB1-41 | NA | NA | -0.67 | -0.80 | -0.82 | NA | -0.63 | NA | -0.67 | -0.62 |
| ASV144_Verrucomicrobiota_WCHB1-41 | NA | NA | NA | 0.65 | 0.65 | NA | NA | NA | 0.84 | 0.62 |
| ASV161_Verrucomicrobiota_WCHB1-41 | NA | NA | NA | 0.64 | 0.67 | NA | NA | NA | 0.69 | NA |
| ASV285_Verrucomicrobiota_WCHB1-41 | NA | NA | NA | 0.70 | 0.72 | NA | NA | NA | 0.80 | NA |
| ASV333_Verrucomicrobiota_WCHB1-41 | NA | NA | NA | 0.62 | 0.62 | NA | NA | NA | 0.71 | NA |
| ASV94_Verrucomicrobiota_WCHB1-41 | NA | NA | NA | 0.70 | 0.74 | NA | NA | NA | 0.80 | NA |
