# Supplementary figures and images for: Temporal dynamics of volatile fatty acids profile, methane production, and prokaryotic community in an in vitro rumen fermentation system fed with maize silage
Source: Front Microbiol. 2024 Feb 20;15:1271599. doi: 10.3389/fmicb.2024.1271599 (PMC10912478; doi:10.3389/fmicb.2024.1271599)

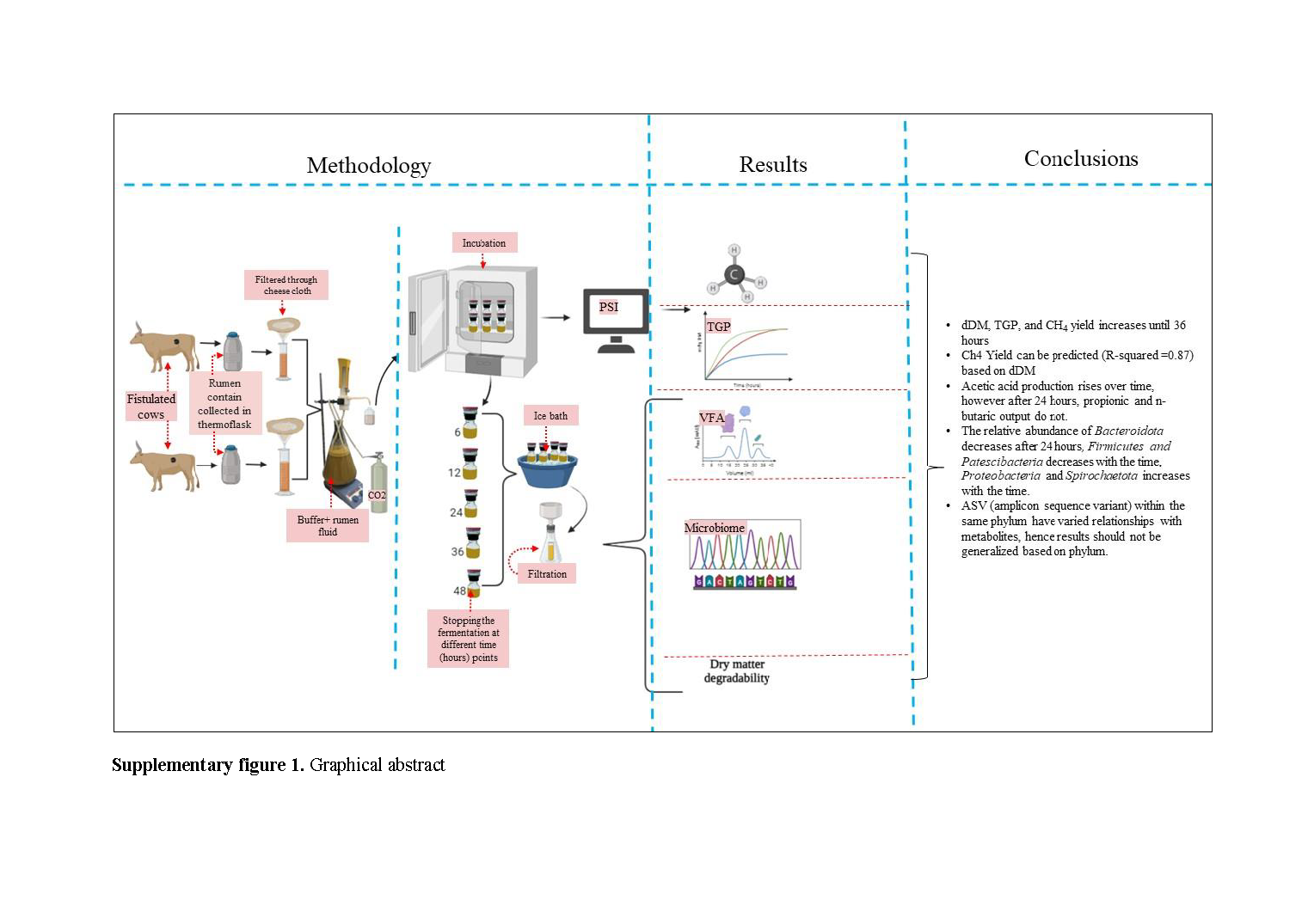

Supplement: Supplementary file 2 [file Image_1.TIF]
